# Epigenome-wide association study identifies DNA methylation loci associated with handgrip strength in Chinese monozygotic twins

**DOI:** 10.3389/fcell.2024.1378680

**Published:** 2024-04-03

**Authors:** Jia Luo, Weijing Wang, Jingxian Li, Haiping Duan, Chunsheng Xu, Xiaocao Tian, Dongfeng Zhang

**Affiliations:** ^1^ Department of Epidemiology and Health Statistics, School of Public Health, Qingdao University, Qingdao, Shandong, China; ^2^ Qingdao Municipal Centre for Disease Control and Prevention, Qingdao, Shandong, China; ^3^ Qingdao Institute of Preventive Medicine, Qingdao, Shandong, China

**Keywords:** epigenetics, DNA methylation, monozygotic twins, handgrip strength, causal inference

## Abstract

**Background:** The decline in muscle strength and function with aging is well recognized, but remains poorly characterized at the molecular level. Here, we report the epigenetic relationship between genome-wide DNA methylation and handgrip strength (HGS) among Chinese monozygotic (MZ) twins.

**Methods:** DNA methylation (DNAm) profiling was conducted in whole blood samples through Reduced Representation Bisulfite Sequencing method. Generalized estimating equation was applied to regress the DNAm of each CpG with HGS. The Genomic Regions Enrichment of Annotations Tool was used to perform enrichment analysis. Differentially methylated regions (DMRs) were detected using *comb-p*. Causal inference was performed using Inference about Causation through Examination of Familial Confounding method. Finally, we validated candidate CpGs in community residents.

**Results:** We identified 25 CpGs reaching genome-wide significance level. These CpGs located in 9 genes, especially *FBLN1*, *RXRA*, and *ABHD14B*. Many enriched terms highlighted calcium channels, neuromuscular junctions, and skeletal muscle organ development. We identified 21 DMRs of HGS, with several DMRs within *FBLN1*, *SLC30A8*, *CST3*, and *SOCS3*. Causal inference indicated that the DNAm of 16 top CpGs within *FBLN1*, *RXRA*, *ABHD14B*, *MFSD6*, and *TYW1B* might influence HGS, while HGS influenced DNAm at two CpGs within *FBLN1* and *RXRA*. In validation analysis, methylation levels of six CpGs mapped to *FLBN1* and one CpG mapped to *ABHD14B* were negatively associated with HGS weakness in community population.

**Conclusion:** Our study identified multiple DNAm variants potentially related to HGS, especially CpGs within *FBLN1* and *ABHD14B*. These findings provide new clues to the epigenetic modification underlying muscle strength decline.

## 1 Introduction

Muscle strength is a crucial factor in healthy aging and an important predictor of several adverse health outcomes, which decline from midlife and throughout life (McLeod et al., 2016; Balogun et al., 2019; Alcazar et al., 2020). HGS, a simple measure of upper body muscle strength in clinical practice (Lauretani et al., 2003), is influenced by both environmental and genetic factors (Tian et al., 2017). The correlation between HGS and various factors, encompassing nutritional parameters and levels of physical activity, has been extensively explored through epidemiological studies (Rondanelli et al., 2016; Mendes et al., 2017; Weng et al., 2022). Moreover, previous genome-wide association studies (GWAS) have elucidated the influence of genetics on HGS, revealing heritability estimates within the range of 40%–65% (Tiainen et al., 2004; Matteini et al., 2010; Matteini et al., 2016). Recent emerging GWASs have facilitated the identification of human genetic variants associated with HGS (Matteini et al., 2016; Luo et al., 2022). Nevertheless, the single nucleotide-level polymorphisms (SNPs) reported in these studies only explained a small portion of heritability of HGS, suggesting that additional gene-regulatory mechanisms are involved in muscle maintenance or decline (He et al., 2020).

Emerging evidence indicated that epigenetic processes play a crucial role in the development of complex human traits or diseases (Godfrey et al., 2007). Epigenetic modifications serve as potential mediators for the impact of external factors on muscle maintenance or decline, providing a mechanistic framework that integrates environmental and genetic influences. DNA methylation, a well-studied epigenetic modification, provides a novel perspective on the variability of muscle strength. It is widely recognized that DNA methylation is a fundamental mechanism underlying myogenesis (Carrió et al., 2015; Laker and Ryall, 2016). Previous research has suggested that DNAm regulates skeletal muscle differentiation and proliferation by modulating the expression of genes from the myocyte enhancer and myogenic pathway (Bharathy et al., 2013). Several studies have reported a hypermethylated state in skeletal muscle tissue in older subjects compared to younger subjects (Zykovich et al., 2014; Turner et al., 2020). There are multiple epigenome-wide association studies (EWAS) of sarcopenia or muscle mass and strength among people of European ancestry (Livshits et al., 2016; Voisin et al., 2021; Antoun et al., 2022). These studies have identified many CpG loci and differentially methylated regions (DMRs) that may be associated with muscle strength. Nevertheless, three EWASs have tested associations between whole-blood DNAm level and HGS, but no significant association between methylation levels at single CpG and HGS was observed (Bell et al., 2012; Marioni et al., 2015; Soerensen et al., 2019).

The trait-discordant monozygotic twins design is a valuable and powerful design for EWAS that can link epigenetic modification to traits, as the genetic component is perfectly matched within monozygotic twin pairs (Tan et al., 2015). This design also provides an opportunity to identify different DNA methylation patterns triggered by environmental factors. Due to difference in environmental exposures, genetic backgrounds, and lifestyles among different ethnicities, DNAm patterns are likely to exhibit distinctions. Hence, it is necessary to conduct comparable studies using samples from different ethnic groups. Currently, relevant investigation has not been reported in the Chinese population.

## 2 Materials and methods

### 2.1 Participants and study procedures

The study population was a subsample of the Qingdao Twin Registry System. For detailed information on recruitment procedures, please refer to our previous work (Xu et al., 2017). Participants were excluded if they suffered from stroke, cardiovascular disease, and/or tumor, or were unable to complete the examination. Twin pairs with intra-pair HGS difference ≥0.1 kg were chosen for the trait-discordant monozygotic twin design based on our previous experience with similar studies (Wang W. et al., 2021; Wang T. et al., 2021; Wang et al., 2023). A total of 66 HGS-discordant monozygotic twin pairs were included. The Regional Ethics Committee of the Institutional Review Committee of Qingdao CDC approved this study. This study followed the Helsinki Declaration and all participants provided informed consent.

After a fasting period of 10–12 h, the participants completed a questionnaire and physical examination, and a venous blood sample of 10 mL was collected. Handgrip strength was measured using a hand-held dynamometer (WCS-100, Nantong, China). Participants were asked to forcefully squeeze the dynamometer three times for each hand, with the maximum value being used for subsequent analysis.

### 2.2 DNA methylation data

Total DNA was extracted from venous blood samples for reduced representation bisulfite sequencing experiments. In summary, the initial step involved digestion of genomic DNA to produce shorter fragments. Subsequently, the CpG-rich DNA fragments underwent bisulfite conversion. Finally, the resulting cDNA library was sequenced. The resulting raw methylation data encompassed 551,447 CpGs throughout the genome of each individual. The raw reads were mapped to the human Genome Reference Consortium Human Build 37 using *Bismark* (Krueger and Andrews, 2011), and methylation levels were determined by smoothing the data with the *BiSeq* package in R software. We maintained control over the coverage, ensuring it was within the 90% quantile. CpGs with an average methylation *β*-value below 0.01 or those with more than 10 missing observations were excluded. The methylation *β*-values were transformed to M-values by log_2_ transformation (Wang T. et al., 2021).

### 2.3 Cell-type composition estimate

DNAm patterns vary across different cell types. To address the potential confounding effect of cell-type composition on DNAm analysis in whole blood, we used *ReFACTor*, a reference-free, unsupervised method that utilizes principal component analysis to estimate and adjust for cellular heterogeneity (Jaffe and Irizarry, 2014; Rahmani et al., 2016). In our study, we utilized the top five components identified by *ReFACTor* to control the potential effects of cell-type heterogeneity in EWAS analysis.

### 2.4 Gene expression data preparation

A subsample of 12 MZ twin pairs were included in the gene expression analysis. Briefly, total mRNA was extracted from whole peripheral blood. Then the RNA-Seq library was constructed and sequenced to obtain the sequenced data, which was mapped to the human genome by *TopHat*_*2*_ (Kim et al., 2013). The gene expression level was evaluated by FPKM value through *Cufflinks*.

### 2.5 Statistical analysis

#### 2.5.1 Epigenome-wide association analysis

For single CpG analysis, generalized estimating equation (GEE) model was applied to estimate the relationship between DNAm and HGS, with taking the correlation within each twin pair into account and adjusting for sex, age, and cell-type composition. We conducted this analysis using the *geeglm* function of *geepack* package in R software (version 4.1.0). The false discovery rate (FDR) was calculated to correct for multiple testing and genome-wide significance was defined as FDR <0.05. We annotated the identified genomic CpGs to the nearest genes by *biomaRt* package in R software. Causal inference.

#### 2.5.2 Causal inference

The potential causal relationships between genome-wide significant CpGs and HGS were estimated using the ICE FALCON method, a regression-based method for causal inference in twin pair or family design (Li et al., 2005; Li et al., 2020). Estimations of *β*_self_, *β*_co-twin_ and *β*’_self_, *β*’_co-twin_ were calculated by the GEE model, where *β*_co-twi*n*_ is the estimation of family confounding proportion; *β*_self_ represents the overall correlation including family confounding and casual proportion; and *β*’co-twin and *β*’_self_ were the estimations of the full model. If the absolute difference between *β*_self_ and *β*’_self_ is greater than the absolute difference between *β*_co-twin_ and *β*’_co-twin_ (ratio >1.5), it suggests a causal relationship. However, if the association is a result of family confounding, the absolute difference between *β*_self_ and *β*’_self_ would be similar to the absolute difference between *β*_co-twin_ and *β*’_co-twin_.

#### 2.5.3 Differentially methylated region (DMR) analysis

Considering that DNAm of adjacent CpGs may be functionally or spatially linked, exploring genomic regions containing biologically linked DNAm may provide new perspectives compared to the analysis of single CpGs. Hence, we explored the DMRs potentially related to HGS by *comb-p* (Pedersen et al., 2012). The significantly enriched DMRs were identified using the Stouffer–Liptak–Kechris (*slk*) correction method with a significance threshold of *p* < 0.05.

#### 2.5.4 Biological pathway analysis

We performed genomic region enrichment analyses using the Genomic Regions Enrichment of Annotations Tool (GREAT) (McLean et al., 2010). The CpGs that were identified with a *p* < 0.05 were submitted to the GREAT online platform for ontology enrichment. Annotation was conducted based on Genome Reference Consortium Human Build 37, employing the default “basal plus extension” association rule. Enrichment items with FDR<0.05 were deemed statistically significant.

#### 2.5.5 EWAS power estimation

According to a recent computer simulation study on the power of EWAS based on twin designs, for traits with a heritability of 0.6 or higher, the maximal sample size of 63 pairs is required for statistical power exceeding 80% in utilizing trait-discordant twin design (Li et al., 2018; Wang et al., 2023). Compared to ordinary case-control design, twin design can significantly reduce the required sample size. Therefore, our sample size could meet the criteria for statistical power exceeding 80%.

#### 2.5.6 DNA methylation and gene expression analysis

The correlation between top CpGs methylation level and corresponding gene expression level was calculated using Spearman’s rank correlation. In addition, we also queried the position of significant CpGs in NCBI to illustrate the gene structures where these CpGs are located.

#### 2.5.7 Quantitative methylation analysis of *FBLN1* and *ABHD14B*

To validate CpGs located at *FBLN1* and *ABHD14B,* we recruited 117 participants with low HGS (defined as male<28 kg, female<18 kg) and 117 healthy controls from the community. The method for HGS testing is consistent with the aforementioned approach. Participants attended interviews and venous blood samples were collected for DNAm level measurements. Primers for the *FBLN1* and *ABHD14B* genes were designed to cover the majority of the CpGs identified in EWAS. The cleavage products were analyzed using MALDI-TOF mass spectrometry based on MassARRAY System (Bio Miao Biological Technology, Beijing, China). Subsequently, the resulting spectra were processed using the MassARRAY EpiTYPER software (Agena Bioscience, San Diego, California) to determine the methylation ratio. The Wilcoxon rank-sum test was used to compare the DNAm levels of GpGs between the two groups. The association between each CpG and low HGS was evaluated by logistic regression, with adjusting for age, sex, and BMI. The significance level was defined as *p* < 0.05.

## 3 Results

The epigenome-wide association study included 66 MZ with a median HGS of 32.0 kg (95% range 16.7, 57.0) and the median of absolute values of intra-pair HGS was 3.9 kg (95% range 0.3, 13.3). Significant correlations were observed between various clinical indicators within twin pairs, such as height, weight, BMI, fat rate, systolic and diastolic blood pressure, glucose, serum uric acid, cholesterol, triglycerides, HDL cholesterol, and LDL cholesterol, suggesting that utilizing a trait-discordant monozygotic twin design provided significant advantages (Supplementary Table S1).

### 3.1 Epigenome-wide association analysis

**FIGURE 1:**
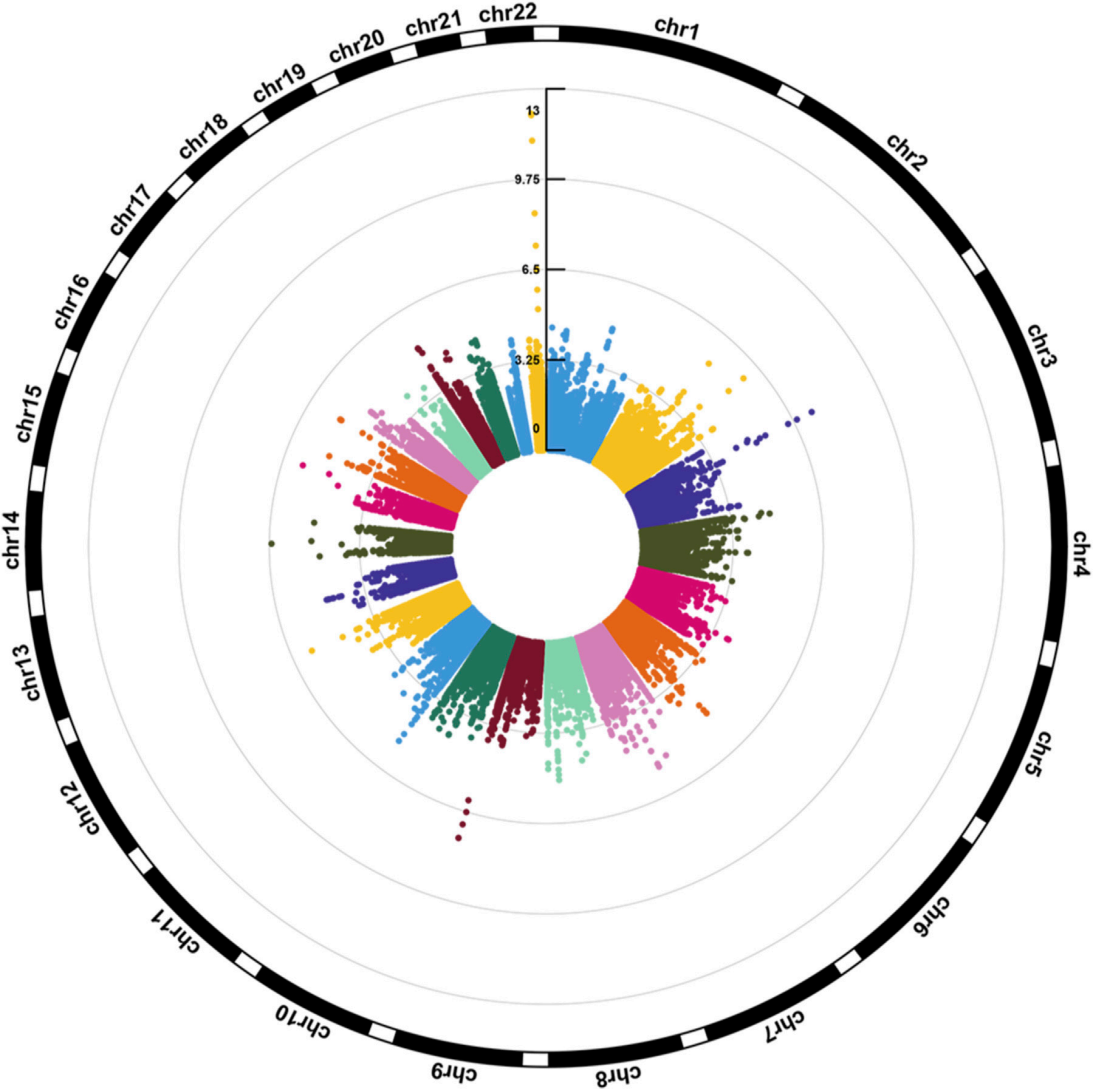
Circular Manhattan plot for the epigenome-wide association study of HGS. The number of chromosomes and the -log_10_ of *p*-values for statistical significance are shown. The dots represent the observed CpGs.

Figure 1 illustrates the Manhattan plot of the EWAS. A total of 25 CpGs were found reaching genome-wide significance level (FDR <0.05). Specifically, eight CpGs (chr3: 45,948,525–45,948,675 bp) located at *FBLN1*; four CpGs (chr9: 137,240,398–137,240,420 bp) located at *RXRA*; four CpGs (chr3: 52,007,520–52,007,647 bp) located at *ABHD14B*, and the remaining nine CpGs located at six different genes, including *ERO1L*, *MFSD6*, *TMEM233*, *TYW1B*, *PAX8*, and *MRPL23*. Methylation level of 16 CpGs (located at *FBLN1*, *ABHD14B*, *MFSD6*, *TMEM233*, and *PAX8* genes) were positively associated with HGS, indicating that hypermethylation of these CpGs in twins with higher HGS. While nine CpGs (located at *RXRA*, *ERO1L*, *TYW1B*, and *MRPL23* genes) were negatively associated with HGS, indicating that hypomethylation of these CpGs in twins with higher HGS (Table 1).

**TABLE 1:** The results of the epigenome-wide association study on HGS (FDR<0.05).

Chr	Position (bp)	Coefficient	*p*-value	FDR	Ensembl gene ID	HGCN symbol
chr22	45,948,613	0.042	7.63E-13	7.18E-08	ENSG00000077942	*FBLN1*
chr22	45,948,616	0.042	8.02E-13	7.18E-08	ENSG00000077942	*FBLN1*
chr22	45,948,620	0.042	8.74E-13	7.18E-08	ENSG00000077942	*FBLN1*
chr22	45,948,590	0.041	7.16E-12	4.41E-07	ENSG00000077942	*FBLN1*
chr22	45,948,556	0.039	2.98E-09	1.47E-04	ENSG00000077942	*FBLN1*
chr9	137,240,415	−0.035	3.27E-08	1.17E-03	ENSG00000186350	*RXRA*
chr9	137,240,420	−0.035	3.31E-08	1.17E-03	ENSG00000186350	*RXRA*
chr22	45,948,659	0.048	4.33E-08	1.33E-03	ENSG00000077942	*FBLN1*
chr3	52,007,647	0.311	5.84E-08	1.60E-03	ENSG00000114779	*ABHD14B*
chr9	137,240,405	−0.034	1.07E-07	2.63E-03	ENSG00000186350	*RXRA*
chr3	52,007,565	0.284	2.29E-07	5.14E-03	ENSG00000114779	*ABHD14B*
chr9	137,240,398	−0.034	3.09E-07	5.85E-03	ENSG00000186350	*RXRA*
chr22	45,948,675	0.059	3.09E-07	5.85E-03	ENSG00000077942	*FBLN1*
chr14	53,131,877	−0.269	3.83E-07	6.75E-03	ENSG00000197930	*ERO1L*
chr3	52,007,520	0.269	5.21E-07	8.56E-03	ENSG00000114779	*ABHD14B*
chr9	140,117,008	0.223	8.26E-07	1.27E-02	NA	NA
chr2	191,295,760	0.250	1.40E-06	2.03E-02	ENSG00000151690	*MFSD6*
chr22	45,948,525	0.035	1.67E-06	2.14E-02	ENSG00000077942	*FBLN1*
chr15	91,369,952	−0.163	1.72E-06	2.14E-02	NA	NA
chr12	120,032,854	0.020	1.74E-06	2.14E-02	ENSG00000224982	*TMEM233*
chr7	72,285,112	−0.222	3.70E-06	4.35E-02	ENSG00000254184	*TYW1B*
chr3	52,007,573	0.244	4.32E-06	4.84E-02	ENSG00000114779	*ABHD14B*
chr2	114,035,021	0.036	4.64E-06	4.85E-02	ENSG00000125618	*PAX8*
chr7	72,285,120	−0.221	4.88E-06	4.85E-02	ENSG00000254184	*TYW1B*
chr11	1,993,029	−0.123	4.92E-06	4.85E-02	ENSG00000214026	*MRPL23*

FDR: false discovery rate; NA, not available.

### 3.2 Causal inference analysis

Table 2 presents the estimation of the causal inference between genome-wide significant CpGs and HGS. In the causal inference from DNAm to HGS, the causal effect was supported for 16 CpGs located at *FBLN1, RXRA, ABHD14B, MFSD6,* and *TYW1B*. The causal effects from HGS to DNAm were also observed among two CpGs located at *FBLN1* and *RXRA*.

**TABLE 2:** Causal inference analysis between CpGs and handgrip strength.

CpG	Chr^b^	HGCN symbol	Position	Methylation to HGS					HGS to methylation				
				*β*_co-twin change_	*P*_co-twin change_	*β*_self-change_	*P*_self-change_	Ratio^a^	*β*_co-twin change_	*P*_co-twin change_	*β*_self-change_	*P*_self-change_	Ratio^a^
1	chr22	*FBLN1*	45,948,590	1.519	2.190E-02	0.044	9.44E-01	34.42	−0.011	1.27E-01	0.008	3.59E-01	-
2	chr22	*FBLN1*	45,948,556	1.539	5.362E-03	0.309	5.49E-01	4.98	−0.011	5.90E-02	0.007	1.92E-01	-
3	chr9	*RXRA*	137,240,415	−2.215	1.210E-13	−0.986	5.76E-06	2.25	0.016	4.98E-02	−0.010	1.63E-01	-
4	chr9	*RXRA*	137,240,420	−2.244	1.300E-14	−0.989	1.24E-06	2.27	0.017	3.86E-01	−0.010	8.26E-01	1.61
5	chr22	*FBLN1*	45,948,659	1.348	1.343E-03	0.018	9.60E-01	73.84	−0.016	9.04E-02	0.011	2.78E-01	-
6	chr9	*RXRA*	137,240,405	−2.025	1.880E-09	−0.864	1.53E-03	2.34	0.015	1.60E-01	−0.009	6.29E-01	-
7	chr3	*ABHD14B*	52,007,565	0.191	6.700E-06	0.106	6.12E-03	1.80	−0.101	1.28E-01	0.037	3.68E-01	-
8	chr9	*RXRA*	137,240,398	−1.827	5.350E-06	−0.730	3.59E-02	2.50	0.014	2.85E-02	−0.007	1.24E-01	-
9	chr22	*FBLN1*	45,948,675	1.628	1.290E-19	0.629	4.53E-07	2.59	−0.023	1.87E-01	0.014	5.96E-01	1.62
10	chr3	*ABHD14B*	52,007,520	0.176	1.370E-04	0.087	4.34E-02	2.02	−0.099	9.60E-02	0.042	3.90E-01	-
11	chr9	*-*	140,117,008	0.263	4.720E-14	0.141	1.07E-07	1.86	−0.102	2.50E-01	0.056	6.52E-01	-
12	chr2	*MFSD6*	191,295,760	0.200	4.859E-02	0.070	4.83E-01	2.87	−0.090	1.98E-01	0.038	3.99E-01	-
13	chr15	-	91,369,952	−0.180	1.060E-10	0.017	4.73E-01	10.42	0.090	3.75E-01	−0.079	6.97E-01	-
14	chr7	*TYW1B*	72,285,112	−0.208	1.960E-08	−0.057	6.07E-02	3.66	0.100	5.23E-01	−0.078	8.68E-01	-
15	chr3	*ABHD14B*	52,007,573	0.132	1.428E-02	0.057	2.71E-01	2.31	−0.063	2.84E-01	0.028	7.30E-01	-
16	chr7	*TYW1B*	72,285,120	−0.210	1.050E-08	−0.059	4.58E-02	3.56	0.100	4.41E-01	−0.077	7.08E-01	-

^a^ Ratio was expressed in absolute value,

^b^ chromosome, HGS, handgrip strength.

### 3.3 Region-based analysis

**FIGURE 2:**
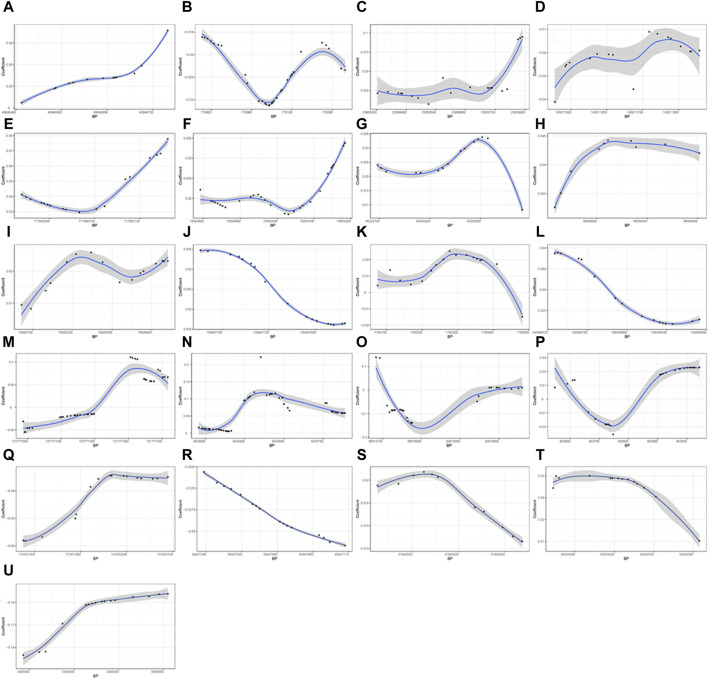
Differential methylation patterns for the identified DMRs. 11 DMRs **(A, C, E–J, L, S, and T)** indicated a positive correlation with HGS, whereas five **(B, D, Q, R, and U)** exhibited a negative association The vertical axis shows the coefficient for the association of each CpG with HGS, and the horizontal axis shows the chromosome positions with the black points indicating each CpG. The black line presents the methylation pattern for each DMR. BP, base pair; chr, chromosome.

A total of 21 DMRs were detected for HGS (Table 3). Notably, among these DMRs, 11 DMRs (A, C, E, F-J, L, S, and T) indicated a positive correlation with HGS, whereas five (B, D, Q, R, and U) near/at *CPLX1*, *PLEC*, *AKR1D1*, *WNK2*, and *ZNF597* exhibited a negative association. In the case of five DMRs (K, M-P), the methylation levels were inconclusive (Figure 2).

**TABLE 3:** The results of annotation to significant differentially methylated regions (DMRs) (*slk* corrected *p* < 0.05).

DMR ID	Chromosome	Start	End	Length	*slk* corrected *p*-value	Gene symbol	Location
A	chr22	45,948,412	45,948,733	13	8.08E-14	*FBLN1*	At
B	chr4	778,877	779,232	31	1.11E-03	*CPLX1*	At
C	chr20	23,636,303	23,636,792	20	1.17E-03	*CST3*	Near
D	chr8	145,011,455	145,011,859	17	1.20E-03	*PLEC*	At
E	chr8	117,962,019	117,962,182	18	1.34E-03	*SLC30A8*	Near
F	chr17	76,354,791	76,355,185	28	1.39E-03	*SOCS3*	At
G	chr21	45,232,096	45,232,383	15	7.28E-03	*AATBC*	At
H	chr1	38,456,158	38,456,303	10	8.03E-03	*SF3A3*	Near
I	chr17	76,880,072	76,880,441	16	9.52E-03	*TIMP2*	At
J	chr6	108,497,677	108,497,832	17	9.96E-03	*NR2E1*	At
K	chr11	1,780,075	1,780,482	17	1.40E-02	*CTSD*	At
L	chr7	149,389,727	149,389,874	16	1.73E-02	*KRBA1*	Near
M	chr10	131,770,987	131,771,418	36	1.90E-02	*EBF3*	Near
N	chr19	4,542,957	4,543,889	52	2.14E-02	*SEMA6B*	At
O	chr16	30,615,708	30,616,651	31	2.25E-02	*ZNFf689*	At
P	chr19	852,547	853,044	25	2.46E-02	*ELANE*	At
Q	chr7	137,831,775	137,832,090	16	2.69E-02	*AKR1D1*	Near
R	chr9	95,947,588	95,947,708	16	2.93E-02	*WNK2*	At
S	chr15	37,402,530	37,402,656	11	2.98E-02	*WDR70*	At
T	chr13	58,204,218	58,204,367	14	3.37E-02	*PCDH17*	Near
U	chr16	3,493,343	3,493,506	16	4.84E-02	*ZNF597*	At

*Slk*, Stouffer-Liptak-Kechris.

### 3.4 Biological pathway analysis

The GO-enriched terms mainly highlighted calcium channels, neuromuscular junctions, and skeletal muscle organ development. Moreover, the pathway terms associated with HGS were significantly enriched, including calcium transport, choline biosynthesis, genes involved in the integration of energy metabolism, PKC-catalyzed phosphorylation of inhibitory phosphoprotein of myosin phosphatase, and the Notch signaling pathway (Table 4). Some items are related to skeletal muscle function in previous studies and have shown a potential role of epigenetic changes in the denervation of calcium channels and neuromuscular junctions.

**TABLE 4:** The top GREAT ontology enrichment for regions potentially related to handgrip strength by using a binomial test.

Ontology database	Term name	Binom FDR Q-value	Binom region fold enrichment
*GO function*			
GO-MF	calcium channel activity	2.84E-18	1.60
GO-MF	inositol trisphosphate phosphatase activity	4.05E-15	5.18
GO-MF	chondroitin sulfate binding	5.58E-13	5.69
GO-MF	calcium ion transmembrane transporter activity	2.62E-12	1.45
GO-MF	vitamin D receptor binding	4.55E-12	2.95
GO-MF	myosin II binding	7.83E-04	4.19
GO-BP	negative regulation of phosphatidylinositol biosynthetic process	2.98E-42	1.36
GO-BP	positive regulation of synaptic growth at neuromuscular junction	5.33E-42	7.74
GO-BP	osteoclast development	7.16E-33	1.14
GO-BP	reduction of endoplasmic reticulum calcium ion concentration	3.68E-20	1.04
GO-BP	positive regulation of tendon cell differentiation	1.05E-18	1.14
GO-BP	regulation of synaptic growth at neuromuscular junction	1.32E-18	7.74
GO-BP	negative regulation of myotube differentiation	2.01E-17	3.70
GO-BP	actin filament bundle assembly	2.86E-17	2.24
GO-BP	skeletal muscle organ development	6.63E-17	1.55
GO-BP	actin crosslink formation	7.63E-15	4.88
GO-BP	calcium ion transmembrane transport	6.88E-14	1.52
GO-BP	skeletal system morphogenesis	1.08E-13	1.36
GO-BP	skeletal muscle tissue development	5.12E-13	1.49
GO-CC	actomyosin	7.81E-33	2.42
GO-CC	actin filament bundle	2.46E-22	2.26
GO-CC	sarcolemma	5.98E-06	1.32
GO-CC	voltage-gated calcium channel complex	3.53E-05	1.52
GO-CC	sarcoplasm	6.59E-04	1.33
GO-CC	troponin complex	1.00E-02	3.59
*Pathways*			
PANTHER	Pentose phosphate pathway	6.29E-12	7.42
PANTHER	Notch signaling pathway	1.49E-06	1.70
PANTHER	GABA-B receptor II signaling	4.07E-06	1.64
PANTHER	Inflammation mediated by chemokine and cytokine signaling pathway	3.36E-05	1.27
PANTHER	Adrenaline and noradrenaline biosynthesis	5.40E-05	1.82
PANTHER	Beta3 adrenergic receptor signaling pathway	5.20E-03	1.56
BioCyc	calcium transports I	9.69E-03	2.41
BioCyc	choline biosynthesis III	1.87E-02	2.07
BioCyc	Triacylglycerol biosynthesis	3.22E-14	2.17
MSigDB	PKC-catalyzed phosphorylation of inhibitory phosphoprotein of myosin phosphatase	1.93E-15	2.21
MSigDB	Notch signaling pathway	2.14E-42	3.18
MSigDB	Control of Gene Expression by Vitamin D Receptor	2.49E-13	3.18
MSigDB	Genes involved in Integration of energy metabolism	8.13E-13	1.54
MSigDB	Genes involved in Pre-NOTCH Transcription and Translation	5.81E-08	1.89
MSigDB	Cytokines and Inflammatory Response	3.80E-02	1.35
MSigDB	Oxidative Stress Induced Gene Expression Via Nrf2	3.90E-02	1.56

GO, gene ontology; BP, biological process; MF, molecular function; CC, cellular component

### 3.5 DNA methylation and gene expression analysis

A total of 12 twin pairs with a median age of 53 years (95% range 43–65) and a median HGS of 36.9 kg (95% range 18.4–61.3) were included in the analysis. Supplementary Table S2 presented the results of DNA methylation and gene expression analysis. Methylation level of CpGs located at *FBLN1*, *RXRA*, and *MRPL23* gene were positively associated with corresponding gene expression level. Methylation level of CpGs located at *ABHD14B* were negatively associated with gene expression. As shown in Supplementary Figure S1, all of these CpGs are located within gene bodies.

### 3.6 Quantitative methylation analysis of *FBLN1* and *ABHD14B*

We quantified nine CpGs (FDR< 0.05) mapped to *FBLN1* using the Sequenom MassARRAY platform. Six CpGs were negatively associated with HGS weakness (Supplementary Table S3). Four out of the five CpGs mapped on *ABHD14B* were quantified and one CpG (chr3: 52,007,647) were not detected in the validation experiment. Just one CpG (chr3: 52,007,595) was validated to be hypomethylated in the low HGS group (Supplementary Table S3), although this CpG did not reach genome-wide significance in the EWAS analysis (FDR = 0.054).

## 4 Discussion

In the present study, we detected epigenetic variants of handgrip strength using EWAS based on monozygotic twins. Our analysis identified several CpGs, genes, DMRs, and pathways that might elucidate the mechanism of muscle strength change. As additional validation, several candidates CpGs mapped to *FBLN1* and *ABHD14B* were quantified and validated.

Research has indicated that the effects of DNA methylation are associated with its occurrence at different positions in the genome and DNA methylation occurring in gene bodies may be related to gene expression (Aquino et al., 2018). Similarly, we found that the methylation levels of CpGs within *FBLN1*, *RXRA*, and *MRPL23* are positively correlated with gene expression. The methylation of CpGs in *FBLN1* is also positively associated with HGS. Fibulin-1, encoded by *FBLN1*, is an important protein in the structure of elastic fibers and basement membranes of various tissues. Previous studies suggested that Fibulin-1 implicated in tissue organogenesis during myotome development, bone formation, ossification, and digits of the development limbs (de Vega et al., 2009; Bohlega et al., 2014; Cooley et al., 2014). Methylation levels of CpGs located at *RXRA* and *MRPL23* were negatively associated with HGS in EWAS. *RXRA* is a member of the nuclear receptor superfamily involved in lipid, glucose, energy, and hormone metabolism. Evidence from animal experiments indicates that *RXRA* promotes adipogenesis and accelerate fat accumulation (Pan et al., 2023). Previous research reported that the DNAm level at four CpGs located at *RXRA* (chr 9: 136,355,569–136,355,885) was negatively associated with bone mass, which might affect muscle strength (Harvey et al., 2014; Kim et al., 2018). The mechanism underlying the relationship between *MRPL23* and muscle strength remains unclear at present and awaits further investigation in future studies. The methylation levels of CpGs located within the gene body of *ABHD14B* is positively associated with HGS and negatively correlated with gene expression. A previous study found that cortical ABHD14B levels were negatively associated with motor resilience, which reflects muscle endurance and motor function in the elderly (Buchman et al., 2023).

The present study also identified 21 DMRs in genomic regions located near or at 21 genes, of which *FBLN1*, *SLC30A8*, *CST3*, and *SOCS3* have potential biological functions related to muscle strength. Sprouse et al. reported that rs13266634 in *SLC30A8* is associated with higher muscle strength and larger arm skeletal muscle volume (Sprouse et al., 2014). The protein encoded by *CST3* has been implicated as a biomarker for sarcopenia, as measured by the creatinine-to-cystatin C ratio (Tabara et al., 2020; Tabara et al., 2021). *PLEC* encodes a cytoskeletal protein that maintains tissue integrity by regulating intracellular signaling in response to mechanical stimulation (Rice et al., 2019). *SOCS3* has been identified as a crucial mediator in myogenesis. *SOCS3* overexpression during myogenesis enhanced myogenin and α-actin mRNA expression (Caldow et al., 2011). The protein encoded by *CTSD* maintains cellular protein homeostasis and is regarded as a bone-related biomarker of osteoporosis (Deng et al., 2021; Wang et al., 2022; Wu et al., 2022).

Our findings provide evidence of causation underlying DNAm-HGS. We observed HGS might be a response to the methylation level of several CpGs located at different genes. The potential function of *FBLN1*, *RXRA*, and *ABHD14B* in muscle strength has been discussed above. Recent research has indicated that the methylation level of *RXRA* promoters could change in response to the external stimulus of mechanical loading (Krstic et al., 2022), which might explain the reason for the DNAm response to HGS changes. Additionally, studies have reported an association between lower plasma vitamin D levels and elevated *RXRA* gene methylation (Curtis et al., 2019; Krstic et al., 2022). Considering the role of vitamin D in muscle strength, the changes in HGS-induced *RXRA* DNA methylation alterations may also be attributed to the potential confounding effect of vitamin D.

The strengths and advantages of the current study are as follows. First, our study included monozygotic twin pairs, which controlled the genetic background and enhanced the credibility of the results. Second, we investigated the causal relationship between DNAm and HGS, providing a deeper understanding of the effect of epigenetic modifications on HGS. Thirdly, our study is the first EWAS of HGS in the Asian population. Given the diverse genetic backgrounds and environmental exposures among different ethnic groups, our research illuminates the intrinsic physiological mechanisms contributing to the variation in HGS.

There are limitations of our study that must be acknowledged. First, our study did not consider physical activity and exercise due to a lack of relevant information, which can directly affect HGS or DNA methylation. The second limitation lies in the use of DNA methylation data derived from blood rather than the more intuitively relevant muscle tissue. Despite the challenges in obtaining muscle tissue, we acknowledge the tissue specificity of DNA methylation. Previous study has also indicated differences in the epigenetic feature between muscle tissue and blood (Slieker et al., 2013). However, a study conducted by Pilling et al. identified potential gene expression characteristics related to muscle strength in the blood and revealed several genes previously reported to be associated with muscle strength (Pilling et al., 2016). Additionally, some studies suggest that while the use of whole blood DNA methylation data may only reflect changes in blood, it can still provide information about variations in muscle mass (Livshits et al., 2016). Therefore, the use of blood samples for epigenetic studies on muscle strength may be a feasible approach (Soerensen et al., 2019). Finally, we recognize that HGS serves as an indicator of upper limb strength rather than a comprehensive measure of whole-body strength. Hence, future research should employ more representative indicators to validate our findings.

## 5 Conclusion

In summary, our study utilizing monozygotic twin pairs detected many DNAm variants that may be associated with handgrip strength, particularly the loci within *FBLN1* and *ABHD14B*. Our findings provide new clues to the epigenetic modification underlying muscle strength.

## Data Availability

The data analyzed in this study is subject to the following licenses/restrictions: We are preparing the raw data (fastq files for RRBS libraries) for uploading to the public website, and until then the data used or analyzed during the current study are available from the corresponding author upon reasonable request. Requests to access these datasets should be directed to DZ, zhangdf1961@126.com.

## Abbreviations

MZ, Monozygotic; EWAS, Epigenome-Wide Association Study; GEE, Generalized estimating equation; ICE FALCON, Inference about Causation through Examination of Familial Confounding method; FDR, False discovery rate; GO, Gene Ontology; KEGG, Kyoto Encyclopedia of Genes and Genomes; DMRs, Differentially methylation regions; HGS, Handgrip strength; DNAm, DNA methylation; GWAS, Genome-wide association study; GREAT, Genomic Regions Enrichment of Annotations Tool.
