# Supplementary material for: Epigenome-wide association study identifies DNA methylation loci associated with handgrip strength in Chinese monozygotic twins
Source: Front Cell Dev Biol. 2024 Apr 3;12:1378680. doi: 10.3389/fcell.2024.1378680 (PMC11021642; doi:10.3389/fcell.2024.1378680)
Supplement: Supplementary file 1 [file Table1.DOCX]

**Supplementary Table 1.** Basic characteristics of the participants

| **Characteristics** | **Values** | **Intrapair correlation** | |
| --- | --- | --- | --- |
|  |  | **r** | ***P*-value** |
| Number of twin pairs | 66 |  |  |
| Gender, pairs |  |  |  |
| Male | 36 | - | *-* |
| Female | 30 | - | *-* |
| Age, mean (SD) (year) | 52.0 (7.4) | - | *-* |
| HGS, M (*P*_2.5_, *P_9_*_7.5_) (Kg) | 32.0 (16.7, 57.0) | 0.86 | *P*<0.001 |
| BMI, mean (SD) (Kg/m^2^) | 25.0 (3.50) | 0.61 | *P*<0.001 |
| High, mean (SD) (cm) | 161.20 (6.52) | 0.91 | *P*<0.001 |
| Weight, mean (SD) (Kg) | 65.0 (10.0) | 0.66 | *P*<0.001 |
| Fat rate, M (*P*_2.5_, *P*_97.5_) (%) | 29.4 (13.1, 48.7) | 0.77 | *P*<0.001 |
| Systolic, M (*P*_2.5_, *P_9_*_7.5_) (mmHg) | 130 (102, 184) | 0.39 | *P*<0.001 |
| Diastolic, M (*P*_2.5_, *P_9_*_7.5_) (mmHg) | 82 (62, 105) | 0.25 | *P*<0.001 |
| SUA, M (*P*_2.5_, *P_9_*_7.5_) (μmol/L) | 286 (163, 520) | 0.47 | *P*<0.001 |
| GLU, M (*P*_2.5_, *P_9_*_7.5_) (mmol/L) | 5.5 (3.83, 10.53) | 0.55 | *P*<0.001 |
| CHOL, M (*P*_2.5_, *P_9_*_7.5_) (mmol/L) | 5.03 (2.71, 6.73) | 0.60 | *P*<0.001 |
| TG, M (*P*_2.5_, *P_9_*_7.5_) (mmol/L) | 1.15 (0.20, 5.65) | 0.59 | *P*<0.001 |
| HDLC, M (*P*_2.5_, *P_9_*_7.5_) (mmol/L) | 1.32 (0.67, 2.56) | 0.76 | *P*<0.001 |
| LDLC, M (*P*_2.5_, *P_9_*_7.5_) (mmol/L) | 2.82 (1.35, 4.40) | 0.49 | *P*<0.001 |

Continuous variables were presented as mean (standard deviation) or median (P_2.5_, P_97.5_); Categorical variables were presented as numbers with percentiles. BMI: body mass index; SUA: serum uric acid; GLU: fasting glucose; CHOL: total cholesterol; TG: triglyceride; HDLC: high-density lipoprotein; LDLC: low-density lipoprotein

**Supplementary Table 2.** The association between gene expressions and DNA methylation

| Chromosome | Position | Gene | r | *P* |
| --- | --- | --- | --- | --- |
| chr11 | 1993029 | *MRPL23* | 0.49 | 0.029 |
| chr22 | 45948525 | *FBLN1* | 0.54 | 0.024 |
| chr22 | 45948556 | *FBLN1* | 0.59 | 0.013 |
| chr22 | 45948590 | *FBLN1* | 0.69 | 0.002 |
| chr22 | 45948613 | *FBLN1* | 0.65 | 0.005 |
| chr22 | 45948616 | *FBLN1* | 0.63 | 0.006 |
| chr22 | 45948620 | *FBLN1* | 0.61 | 0.009 |
| chr22 | 45948659 | *FBLN1* | 0.17 | 0.505 |
| chr22 | 45948675 | *FBLN1* | 0.05 | 0.842 |
| chr3 | 52007520 | *ABHD14B* | -0.62 | 0.003 |
| chr3 | 52007565 | *ABHD14B* | -0.62 | 0.003 |
| chr3 | 52007573 | *ABHD14B* | -0.61 | 0.004 |
| chr3 | 52007647 | *ABHD14B* | -0.59 | 0.005 |
| chr14 | 53131877 | *ERO1L* | / | / |
| chr7 | 72285112 | *TYW1B* | / | / |
| chr7 | 72285120 | *TYW1B* | / | / |
| chr15 | 91369952 | *NA* | / | / |
| chr2 | 114035021 | *PAX8* | 0.37 | 0.111 |
| chr12 | 120032854 | *TMEM233* | -0.05 | 0.845 |
| chr9 | 137240398 | *RXRA* | 0.55 | 0.011 |
| chr9 | 137240405 | *RXRA* | 0.57 | 0.008 |
| chr9 | 137240415 | *RXRA* | 0.57 | 0.008 |
| chr9 | 137240420 | *RXRA* | 0.57 | 0.008 |
| chr9 | 140117008 | *NA* | / | / |
| chr2 | 191295760 | *MFSD6* | / | / |

/ indicates that the corresponding CpG sites are not located within any gene or lack expression level data for the respective genes

**Supplementary Table 3.** The results of validation analysis for the CpGs mapped to *FBLN1* on handgrip strength weakness

| **CpG No.** | **Chr** | **Position (bp)** | **Discovery** | | **Validation** | | |
| --- | --- | --- | --- | --- | --- | --- | --- |
|  |  |  | Coefficient | *P*-value | *P*-value of comparison between groups | OR (95% CI) | *P*-value |
| 1 | chr22 | 45948613 | 0.042 | 7.63E-13 | 0.649 | 0.998 (0.956-1.042) | 0.934 |
| 2^*^ | chr22 | 45948616 | 0.042 | 8.02E-13 | 0.032 | 0.596 (0.383-0.888) | 0.017 |
| 3^*§^ | chr22 | 45948620 | 0.042 | 8.74E-13 | 0.001 | 0.493 (0.264-0.871) | 0.02 |
| 4^*§^ | chr22 | 45948590 | 0.041 | 7.16E-12 | 0.001 | 0.493 (0.264-0.871) | 0.02 |
| 5^*§^ | chr22 | 45948556 | 0.039 | 2.98E-09 | 0.001 | 0.493 (0.264-0.871) | 0.02 |
| 6^*†^ | chr22 | 45948659 | 0.048 | 4.33E-08 | 0.001 | 0.245 (0.101-0.554) | 0.001 |
| 7 | chr22 | 45948675 | 0.059 | 3.09E-07 | 0.438 | 0.987 (0.956-1.020) | 0.436 |
| 8^*†^ | chr22 | 45948525 | 0.035 | 1.67E-06 | 0.001 | 0.245 (0.101-0.554) | 0.001 |
| 9 | chr22 | 45948516 | 0.034 | 8.34E-06 | 0.148 | 0.967 (0.821-1.124) | 0.661 |

Note: * The CpG significantly associated with handgrip strength weakness. § Those 3 CpGs were consecutive detection sites, and the methylation level were the average value of the 3 units. †These 2 CpGs were consecutive detection sites, and the methylation level were the average value of the 2 units.

**Supplementary Table 4.** The results of validation analysis for the CpGs mapped to *ABHD14B* on handgrip strength weakness

| **CpG No.** | **Chr** | **Position (bp)** | **Discovery** | | **Validation** | | |
| --- | --- | --- | --- | --- | --- | --- | --- |
|  |  |  | Coefficient | *P*-value | *p*-value of comparison between groups | OR (95% CI) | *P*-value |
| 1^#^ | chr3 | 52007647 | 0.311 | 5.84E-08 | - | - | - |
| 2^†^ | chr3 | 52007565 | 0.284 | 2.29E-07 | 0.229 | 0.992 (0.848-1.155) | 0.918 |
| 3^†^ | chr3 | 52007520 | 0.269 | 5.21E-07 | 0.229 | 0.992 (0.848-1.155) | 0.918 |
| 4 | chr3 | 52007573 | 0.244 | 4.32E-06 | 0.959 | 0.991 (0.952-1.031) | 0.64 |
| 5^*^ | chr3 | 52007595 | 0.24 | 7.16E-06 | 0.043 | 0.954 (0.911-0.999) | 0.045 |

Note: # The CpGs were not detected in the validation experiment. * The CpG significantly associated with handgrip strength weakness. †These 2 CpGs were consecutive detection sites, and the methylation level were the average value of the 2 units.


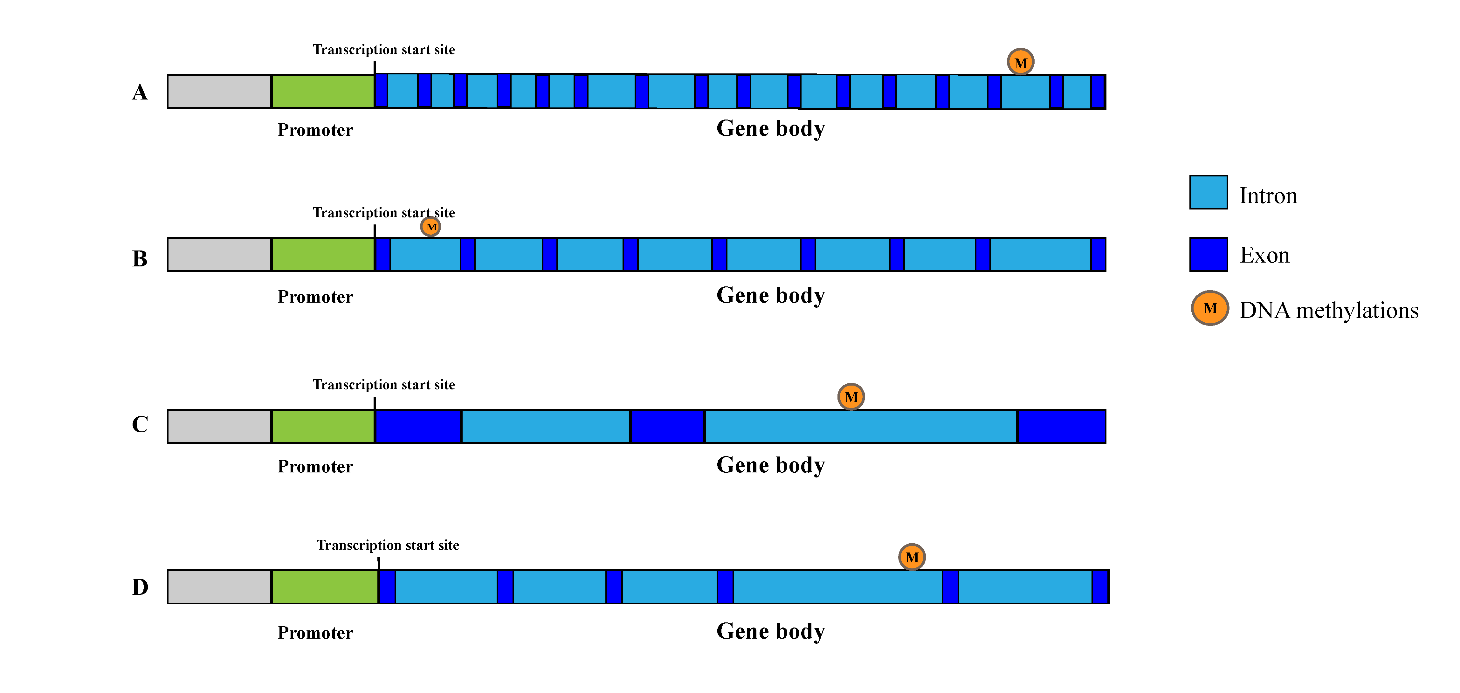


**Figure S1.** Schematic diagram of the top CpGs and corresponding gene structures; (A) *FBLN1* gene, the top CpGs were located at chr22: 45948525-45948675; (B) *RXRA* gene, the top CpGs were located at chr9: 137240398-137240420; (C) *ABHD14B* gene, the top CpGs were located at chr3: 52007520-52007647; (D) *MRPL23* gene, the top CpG was located at chr11: 1993029.
